# Convergence of Prognostic Gene Signatures Suggests Underlying Mechanisms of Human Prostate Cancer Progression

**DOI:** 10.3390/genes11070802

**Published:** 2020-07-16

**Authors:** Bogdan-Alexandru Luca, Vincent Moulton, Christopher Ellis, Shea P. Connell, Daniel S. Brewer, Colin S. Cooper

**Affiliations:** 1 School of Computing Sciences, University of East Anglia, Norwich Research Park, Norwich NR4 7TJ, UK; b.luca@stanford.edu (B.-A.L.); v.moulton@uea.ac.uk (V.M.); c.ellis1@uea.ac.uk (C.E.); 2 Norwich Medical School, University of East Anglia, Norwich Research Park, Norwich NR4 7TJ, UK; s.connell@uea.ac.uk (S.P.C.); d.brewer@uea.ac.uk (D.S.B.); 3 The Earlham Institute, Norwich Research Park, Norwich NR4 7UZ, UK

**Keywords:** prostate cancer, prognostic signature, diagnostic signature, biomarkers, cancer progression, aggressive cancer

## Abstract

The highly heterogeneous clinical course of human prostate cancer has prompted the development of multiple RNA biomarkers and diagnostic tools to predict outcome for individual patients. Biomarker discovery is often unstable with, for example, small changes in discovery dataset configuration resulting in large alterations in biomarker composition. Our hypothesis, which forms the basis of this current study, is that highly significant overlaps occurring between gene signatures obtained using entirely different approaches indicate genes fundamental for controlling cancer progression. For prostate cancer, we found two sets of signatures that had significant overlaps suggesting important genes (*p* < 10^−34^ for paired overlaps, hypergeometrical test). These overlapping signatures defined a core set of genes linking hormone signalling (HES6-AR), cell cycle progression (Prolaris) and a molecular subgroup of patients (PCS1) derived by Non Negative Matrix Factorization (NNMF) of control pathways, together designated as SIG-HES6. The second set (designated SIG-DESNT) consisted of the DESNT diagnostic signature and a second NNMF signature PCS3. Stratifications using SIG-HES6 (HES6, PCS1, Prolaris) and SIG-DESNT (DESNT) classifiers frequently detected the same individual high-risk cancers, indicating that the underlying mechanisms associated with SIG-HES6 and SIG-DESNT may act together to promote aggressive cancer development. We show that the use of combinations of a SIG-HES6 signature together with DESNT substantially increases the ability to predict poor outcome, and we propose a model for prostate cancer development involving co-operation between the SIG-HES6 and SIG-DESNT pathways that has implication for therapeutic design.

## 1. Introduction

A major problem in management of human prostate cancer is the high variability in its clinical course making prediction of outcome at the time of diagnosis or following radical therapy extremely difficult [1,2]. A critical challenge is to improve prediction of outcome beyond the use of standard clinical predictors including D’Amico stratification and CAPRA score [3]. For prostate cancer, the development of expression-based prognostic biomarkers has proven very fruitful with over 20 predictive signatures and classifications reported. Many signatures were derived using supervised approaches involving comparisons of aggressive and nonaggressive disease [4,5,6,7,8,9,10,11,12,13,14,15,16,17]. Several biomarkers represent particular biological functions [18,19,20,21,22]. For example, the Prolaris biomarker [19] contains genes known to be involved in controlling transition through the cell cycle.

Unsupervised approaches may also be used for classification and biomarker identification [23,24,25,26].We have used an unsupervised mathematical approach called Latent Process Decomposition (LPD) that takes into account the issue of heterogeneity within individual prostate samples to identify a new poor prognosis category of prostate cancer called DESNT [23,27]. In an alternative unsupervised approach, the status of control pathways deduced from expression datasets was analysed using Non-Negative Matrix Factorisation (NNMF), leading to the identification of a poor prognosis category called PSC1 [26]. The presence of somatic copy number alterations, sometimes linked to the expression of genes within regions of alteration, has also be utilised for biomarker identification [28].

An interesting feature of biomarkers discovery involving comparisons of expression linked to different clinical states are the small overlaps between different predictive gene lists for the same biological endpoint. This observation, and its underlying causes, are well documented for human breast cancer [29,30,31]. A series of studies demonstrated that the lack of overlap cannot be simply be attributed to trivial reasons such as the use of different patient cohorts, different detection technologies, and different analytical methods. This is illustrated by the work of Ein-Dor et al. [29], who repeated the analysis performed by van’t Veer et al. [32] during their derivation of the Mammaprint 70-gene predictive signature for breast cancer outcome. Using transcriptome data from many subsets of training samples that were selected from the complete van’t Veer et al. dataset, they demonstrated that multiple different but equally predictive 70-gene signatures could be derived. They noted that for many hundreds of individual genes, the correlation with survival had intermediate predictive values and that the differences between values were very small. The relative ranking of genes changed dramatically when slightly different training sets were used, leading to the selection of poorly overlapping predictive signatures.

In the current study, we wished to examine the relationship between biomarker signatures that were derived using a variety different approaches. Our hypothesis is that the progression of prostate cancer occurs via one or a small number of underlying biological process and that the significant overlaps between prognostic signatures obtained by independent methods of discovery and using different datasets may indicate sampling from genes fundamental for controlling cancer progression.

## 2. Materials and Methods

### 2.1. The You et al. Discovery Cohort (DISC)

To repeat the work of You et al., we complied and normalised expression profiles from the same set of 38 public datasets as in You et al. [26] (DISC cohort) except that we did not include the ArrayExpress dataset E-SMDB-2486, which contains the same samples as the GEO dataset GSE3933. Where available, the raw data has been retrieved from GEO and ArrayExpress repositories, otherwise the provided normalised data from the datasets were used. For the TCGA dataset, the RNA-seq level 3 raw expression data have been downloaded from the TCGA data portal. Only the 217 TCGA samples uploaded before 24/04/2013 have been included in the DISC cohort. Two-channel microarray datasets have been internally normalised using the loess method [33] and across arrays using the quantile method [34]—both implemented in the limma R package [35]. One-channel microarrays have been normalised across arrays using the RMA algorithm [36] implemented in either the *affy* [37] or *oligo* [38] R packages, depending on the microarray platform. RNA-seq raw read counts have been processed using the variance stabilizing transformation implemented in DESeq2 [39]. For datasets that contained samples from more than one platform, samples from each platform have been normalised separately. The probes from the three platforms used in the GSE6919 dataset have been merged into a single sample, for each patient id. Probes from each platform have been annotated to Entrez gene identifiers using the corresponding Bioconductor annotation packages, if available; otherwise the probe identifiers have been converted to entrez ids using the SOURCE interface (http://source-search.princeton.edu/help/SOURCE/resultsBatchHelp.html), Agilent annotation lists or biomaRt package [40]. The Multi-Dimensional-Scaling (MDS) decomposition of the expression profiles of the DISC cohort are shown in Figure S1.

For each dataset, duplicate probes for each Entrez Id have been removed, keeping only the probe with the highest mean expression across samples. The gene intensities have been centred by subtracting the median across samples. The DISC cohort has been then assembled by matching the Entrez Ids, resulting in a cohort of 2707 samples and 32,832 genes. Subsequently, the primary tissue samples without an associated Gleason score, have been removed, resulting in a set of 1381 samples. To remove dataset and platform-specific effects, the data was median-centered and the quantile normalised (MCQ) as described in You et al. [26]. Potential differences compared to the original protocol of You et al. may arise because of the removal of sample duplication and the use of the most probable approach when the published protocol was not completely clear.

### 2.2. Validation Datasets

Four publicly available transcriptome microarray datasets derived from prostatectomy samples from men with prostate cancer were used as a validation dataset and are referred to as: Memorial Sloan Kettering Cancer Centre (MSKCC) [41], CancerMap [23], CamCap [24], and SWD [42]. From the MSKCC dataset, only prostatectomy specimens were used, both in the derivation of the original DESNT classification and for validation analyses in the current studies. The CamCap dataset was produced by combining two Illumina HumanHT-12 V4.0 expression beadchip datasets (GEO: GSE70768 and GSE70769) obtained from two prostatectomy series (Cambridge and Stockholm). The original CamCap [24] and CancerMap [23] datasets have 40 patients in common and thus 20 of the common samples were excluded at random from each dataset. Each Affymetrix Exon microarray dataset was normalised using the RMA algorithm [36] implemented in the Affymetrix Expression Console software. For CamCap and Stephenson, previous normalised values were used. The ComBat algorithm from the sva R package and quantile transformation, was used to mitigate series-specific effects.

### 2.3. Replicating You et al. Analysis

#### 2.3.1. Pathway Activation Z-Score

For a given pathway and a given sample, the pathway activation score has been calculated as indicated in Levine et al. [43], namely:

(1) $$Z_{tS}=\frac{{\bar{X}}_{tS}-{\bar{X}}_{t}}{\sigma_{t}}\sqrt{\left|S\right|}$$

where ${\bar{X}}_{tS}$ is the mean expression level of the genes in pathway *S* and sample *t*, ${\bar{X}}_{t}$ is the mean expression level of all genes in sample *t*, $\sigma_{t}$ is the standard deviation of all genes in sample *t*, and |S| is the number of genes in the set *S*.

#### 2.3.2. Non-Negative Matrix Factorization

Non-negative matrix factorization (NNMF) algorithm implemented in the NNMF R package [44] was used with default parameters.

#### 2.3.3. NNMF Random Forest Classifier

A random forest classifier was trained on the DISC cohort to discriminate between the three NNMF clusters, using as features the 14-pathway z-scores calculated as described above. The model has been built using the implementation from the randomForest R package. The number of trees has been set to 5001, and samples within each class are down-sampled to the frequency of the smallest class; otherwise, the default settings have been used. The model obtained an out-of-bag (OOB) overall accuracy of 92.5%, and a per-class AUC of 0.98–0.99 (Figure S2a–c).

### 2.4. Replicating the Ramos-Montoya Classifier

To reproduce the Ramos-Montoya classification, a random forest model has been trained on the MSKCC dataset. It uses as training labels the assignment of the MSKCC samples into two classes available in Figure 4a of Ramos-Montoya et al. [22] and as features, the 222 genes in the Ramos-Montoya signature. The model has been built using the *randomForest* R package. The number of tress has been set to 5001, and samples within each class are down-sampled to the frequency of the smallest class; otherwise, the default settings have been used. The model obtained an out-of-bag (OOB) accuracy of 92.67%, and a per-class AUC of 0.99 (Figure S2d).

### 2.5. Replicating the Prolaris Classifier

For the Prolaris classification on a given sample, a score is calculated by averaging the within-sample *z*-score normalised expression of the 31 CCP genes [19]. For a given dataset, the top 25% of patients with the highest score are considered high-risk.

### 2.6. LPD (Latent Process Decomposition) DESNT

LPD [45,46] is an unsupervised Bayesian approach which breaks down (decomposes) each sample into component sub-elements (signatures). Each signature is a representative gene expression pattern. LPD is able to classify complex data based on the relative representation of these signatures in each sample and can objectively assess the most likely number of signatures. The approach can take into account the heterogeneous composition of individual prostate cancer samples. The LPD procedure was carried out exactly as described previously [23,27]. The OAS-LPD algorithm is a modified version of the LPD algorithm in which new sample(s) are decomposed into LPD signatures, without retraining the model (i.e., without re-estimating the model parameters µ_gk_, σ^2^_gk_, and α). OAS-LPD was carried out exactly as previously described [27].

### 2.7. Statistical Analysis

The statistical analyses have been carried out in R version 3.3.2. For determining the statistical significance of intersection between two sets of genes, the hypergeometrical test has been used. Genes were defined as differentially expressed if the FDR-adjusted *p*-value < 0.001 and a fold change > 1.4 and identified for each comparison using a moderated *t*-test implemented in the limma R package. Gene set enrichment analysis was performed using the *Fast Gene Set Enrichment Analysis* Bioconductor package [47] using 10,000 permutations. Survival analyses were performed using the log-rank test and Kaplan–Meier estimator, as implemented in the *survival* R package with biochemical recurrence after prostatectomy as the end point. All survival analyses were performed on the combined CancerMap, CamCap and MSKCC cancer datasets (*n* = 482) unless otherwise stated.

## 3. Results

### 3.1. Relationships between Prostate Cancer Signatures

**Figure 1:**
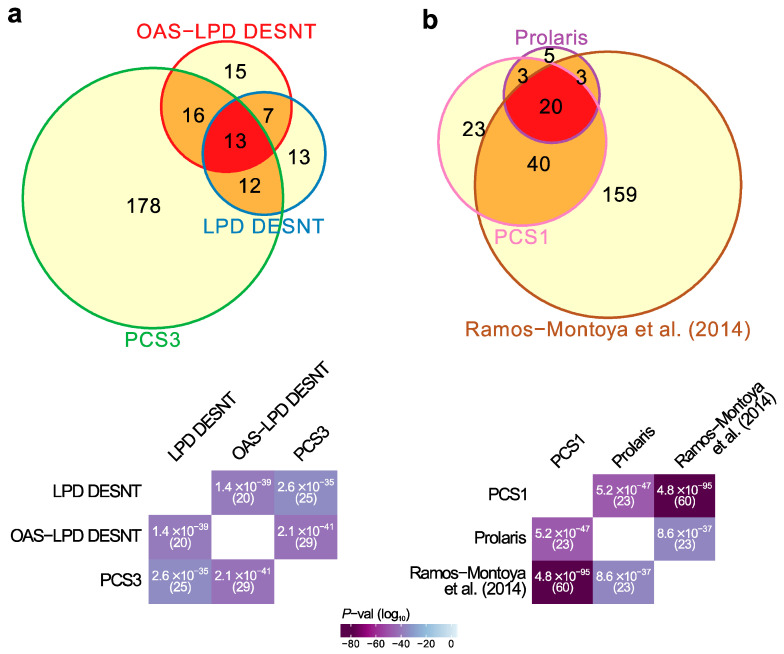
Highly significant signature overlaps. (**a**) Overlaps between LPD DESNT, OAS-LPD DESNT and PCS3. (**b**) Overlap between Prolaris, Ramos-Montoya and PCS1 gene signatures. For each pair of signatures, the probability of the observed overlap occurring by chance was calculated as described in the materials and methods.

As a starting point for this study, we compared 25 published mRNA expression signatures derived to predict aggressive human prostate cancer (Table 1, Data S1). The majority of the gene signatures were determined by comparisons of expression patterns with clinical endpoints, and are predicted to have small overlaps [29]. The pattern of overlaps observed in general fitted this model (Figure S3). We noted two highly significant sets of overlaps (Figure 1a,b, Figure S3, *p* < 10^−34^ for paired overlaps, hypergeometrical test) involving signatures that were derived using unsupervised approaches or that involved investigations of particular biological pathways.

First, there was an overlap between the DESNT genes detected as important by two different LPD procedures, LPD-DESNT [23] and OAS-LPD DESNT [27], and the gene differentially over-expressed in the PCS3 subgroup detected by You et al. [26] (*p* = 2.6 × 10^−35^ and 2.1 × 10^−41^; hypergeometrical test)(Figure 1a, Data S2). In the work of You et al., three groups designated PCS1 (86 genes), PSC2 (123 genes) and PCS3 (219 genes) were detected by non-negative matrix factorisation (NNMF) of the control pathway status calculated from the observed cancer expression profiles. This match could be considered as a match to PSC1 because DESNT genes are under-expressed and the genes overexpressed in PCS3 are also under-expressed in PCS1 [26] (Figure S4). This pathway is referred to as SIG-DESNT and the set of genes within PCS3 that match the DESNT genes is referred to as PCS3-U (U = underexpressed).

Secondly, there was a three-way overlap between the genes associated with PCS1 of You et al. [26], the Prolaris test genes [19], and the signature of Ramos-Montoya et al. [22] (Figure 1b, Data S3). The Prolaris genes were chosen based on their role in controlling cell cycle. Ramos-Montoya et al. selected genes that were controlled by HES6, a transcription factor that has a critical role in driving the androgen receptor (AR) program. This pathway is referred to as SIG-HES6. The 20 genes within PCS1 matching Prolaris and the HES6 signature are all overexpressed. These genes are referred to as PCS1-O (overexpressed) and are distinct from the genes overlapping with the DESNT signature (Data S2).

We were interested to compare cancers detected by these two groups of biomarkers (SIG-DESNT and SIG-HES6) to test whether they are sampling from the same or different cancers at high risk of PSA failure. To examine high risk cancers detected by NNMF of control pathways, we needed first to repeat the analyses carried out by You et al.

### 3.2. Cancer Subgroups Identified by Non-Negative Matrix Factorisation of Control Pathways

**Figure 2:**
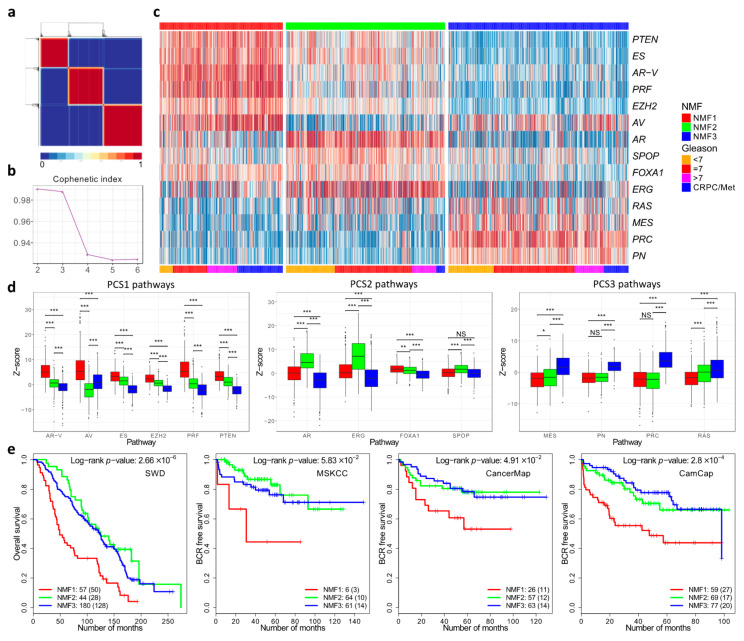
Non-Negative matrix factorisation of control pathways identified three prostate cancer categories. (**a**) Consensus matrix showing three cancer categories. (**b**) Cophenetic coefficient from rank 2 to 6. (**c**) Pathway activation profiles for each cancer (*n* = 1381) arranged according to the three cancer categories NMF1, NMF2 and NMF3. (**d**) The distribution of pathway activation scores within each cluster. The panels correspond to the three groups of pathways that are over-expressed in each cluster in the You et al. paper. (**e**) Kaplan–Meier plots for four different datasets showing clinical outcome for cancers assigned to the three different cancer categories NMF1, NMF2 and NMF3. * ≤0.05; ** ≤0.01; *** ≤0.001.

To repeat the work of You et al. we initially complied and normalised expression profiles from 37 public datasets as outlined by the authors (DISC cohort). This resulted in a combine dataset with linked clinical data consisting of primary prostate cancer (*n* = 1059), non-malignant prostate tissue (*n* = 746), and metastatic samples from men with castration-resistant prostate cancer (*n* = 254) (Materials and Methods, Figure S1). We separately compiled a validation cohort consisting of prostatectomy specimens from the MSKCC, CancerMap, CamCap and SWD datasets. For analysis, we used the 14 pathways that were selected by You at al. on the basis of their likely involvement in prostate cancer development. Scores representing the activation status of each pathway in each sample were aggregated into a *z*-score. Computation of the cophenetic coefficient using a putative number of subgroups between two and six indicated three as the most likely number of subgroups (Figure 2a,b).

Assignment of samples to the three subgroups was carried out by NNMF using a 14xN matrix populated with *z*-scores as a starting point. The results showed that the three subgroups were detected each with a distinct pattern of pathway activation (Figure 2c). The three subgroups were designated NMF1, NMF2 and NMF3. The NMF1 subgroup exhibited overexpression of the AV, AR-V, PRF, PTEN, ES control pathways, while NMF2 had overexpression of the ERG, AR and FOXA1 pathways (Figure 2d). NMF3 was characterised by overexpression of the PRC, PN, MES and RAS pathways. These correspond to the patterns of pathway activation to the three subgroups PCS1, PCS2 and PSC3, respectively, identified by You et.al. The assignment of the majority of the samples (94%) was the same (Table S1). However, because of the small differences, a distinct nomenclature is used in our study (e.g., NMF1 instead of PCS1). The differences may reflect small deviations in the datasets and in the methodology used (Materials and Methods).

We identified 262 genes (NMF1 *n* = 74; NMF2 *n* = 85; NMF3 *n* = 103; FDR < 0.0001; fold-change > 1.4) that were differentially expressed between the three subgroups NMF1, NMF2 and NMF3: 155 of these overlapped with the 428 differentially expressed genes that distinguished PCS1, PCS2 and PCS3 (Figure S5, Data S4). The overlap between the 262 differentially expressed genes and DESNT genes remained highly significant (Data S5): OAS-LPD DESNT and NMF3 (13 genes, *p* = 2.8 × 10^−17^; hypergeometrical test); and DESNT and NMF3 (11 genes, *p* = 2.2 × 10^−14^).

Finally, a random forest classifier trained on the division into NMF1, NMF2, and NMF3 in the original dataset was used to interrogate four test datasets (Figure 2e). In each case, there was a significantly worse outcome for patients assigned to the NMF1 subgroup compared to the NMF2 and NMF3 datasets, consistent with the poor outcome observed for the PCS1 dataset of You et al. We conclude that we have achieved a similar although not identical stratification of cancer samples to that achieved by You et al. and that this may be used as a comparator with DESNT cancer and other stratifications.

### 3.3. Overlaps in the Detection of Cancers at High Risk of PSA Failure

Returning to the comparisons of cancers detected by the SIG-DESNT and SIG-HES6 groups of signatures, we combined data from the CancerMap, CamCap and MSKCC datasets (*n* = 482 patients) with PSA failure as an end point, and then separately applied the DESNT, HES6, NMF1, and Prolaris tests. DESNT is calculated as a continuous variable designated γ representing the proportion of the analysed sample that contains the DESNT signature. Cancers were assigned as a “DESNT cancer” when this gene expression pattern had a larger γ value than any other contributing signatures. Random forest classifiers were used to detect NMF1 and Ramos-Montoya et al. high-risk cancers (Figure S2e–g). We used a published formula to calculate the Prolaris index [19] and selected the 25% of cancers exhibiting highest risk.

Based on the assumption that each of the two signature groups (SIG-DESNT and SIG-HES6) represents a separate underlying molecular mechanism, there are two predicted results. Each signature group could represent an entirely separate progression mechanism in which case two non–overlapping groups of cancers with PSA failures should be detected. Alternatively, the two underlying mechanisms may cooperate to cause cancer progression, meaning that the SIG-DESNT and SIG-HES6 predictors will detect the same or overlapping groups cancers with PSA failure.

**Figure 3:**
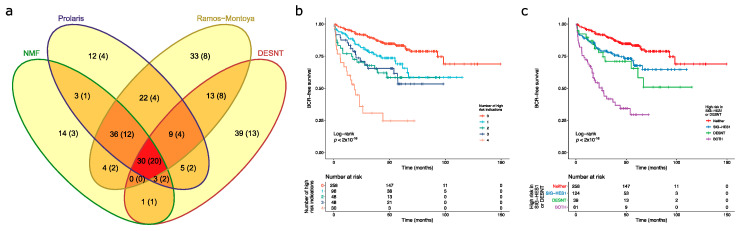
Detection of high-risk cancers. For each sample in the combined dataset obtained by merging the CamCap, CancerMap and MSKCC datasets, we determined whether the patient was deemed high risk using four biomarkers: NMF1, Prolaris, Ramos-Monotoya et al. and DESNT. (**a**) The intersections between the four high-risk categories. The samples in brackets indicate the number of PSA failures. NMF refers to NMF1. (**b**) Kaplan–Meier plot when patients are grouped by the number of biomarkers that indicate that they are high risk. Endpoint is the time to biochemical recurrence. (**c**) Kaplan–Meier plot when patients are grouped by whether they are deemed high risk for DESNT, for at least one of the component biomarkers of SIG-HES6, or for both.

The overlaps in memberships of each group at high risk of PSA failure are shown in Figure 3 supporting the second of these models. A total of 30 cancers were assigned as high risk by all of the prognostic makers and of these 20 had undergone PSA failure (66.7%). Of 100 cancers with 50 PSA failures assigned the SIG-DESNT signature (DESNT), 61(37 PSA failures) were also detected by at least one of the SIG-HES6 signatures (HES6, Prolaris, and/or NMF1) (Figure 3). A combination of the Ramos-Montoya et al. (SIG-HES6) and DESNT (SIG-DESNT) models predicted the majority of PSA failures present in the high-risk cancer groups (76 of 84, 90.5%), with 32 failures overlapping.

Kaplan–Meier analyses were preformed to investigate the interactions between the high-risk categories. A particularly poor outcome was observed for patients designated as high risk by all four biomarkers (Figure 3b). Looking at the interactions between SIG-HES6 and SIG-DESNT biomarkers, we found intermediate rates of progression for patients deemed high risk either for DESNT (*p* = 0.0037 Benjamini–Hochberg adjusted for multiple testing (BH); pairwise comparison between DESNT only and neither; 26.9 vs. 93.0 months to 25% events) or for at least one of the SIG-HES6 biomarkers (Prolaris and/or PCS1 and/or Ramos-Monotoya et al.) (BH *p* = 0.0039; 42.5 vs. 93.0 months to 25% events; Figure 3c). However, when patients were designated as high risk both by DESNT and by at least one of the SIG-HES6 biomarkers, their outcome was considerably worse (Time to 25% events = 8.0 months; BH *p* < 2 × 10^−16^ both vs. neither; Figure 3c). This observation is consistent with our hypothesis that two underlying mechanisms represented by SIG-HES6 and SIG-DESNT are interacting to cause cancer progression.

Upon investigation of whether poor outcome was simply determined by Gleason Score, we found that the number of signatures that indicated that a patient was at high risk was an independent prognostic indicator when Gleason was included as a covariate (IQR HR = 1.98; 95% CI 1.54–2.55; *p* = 1.01 × 10^−7^; Cox proportional hazards regression model). In additional, the combination of a high risk defined by at least one member of SIG-HES6 and DESNT is an independent prognostic indicator when Gleason is included as a covariate [HR = 3.86 (95% CI 2.41–6.19)]. This compares to DESNT only [HR = 1.85 (0.99–3.46)], and SIG-HES6 only [HR = 1.61 (95% CI 1.02–2.52)] (Cox proportional hazards regression models). These results show that designation as high risk provides additional prognostic information to that determined by Gleason Score.

### 3.4. Comparison of DESNT and Non-Negative Matrix Factorisation Subgroups

**Figure 4:**
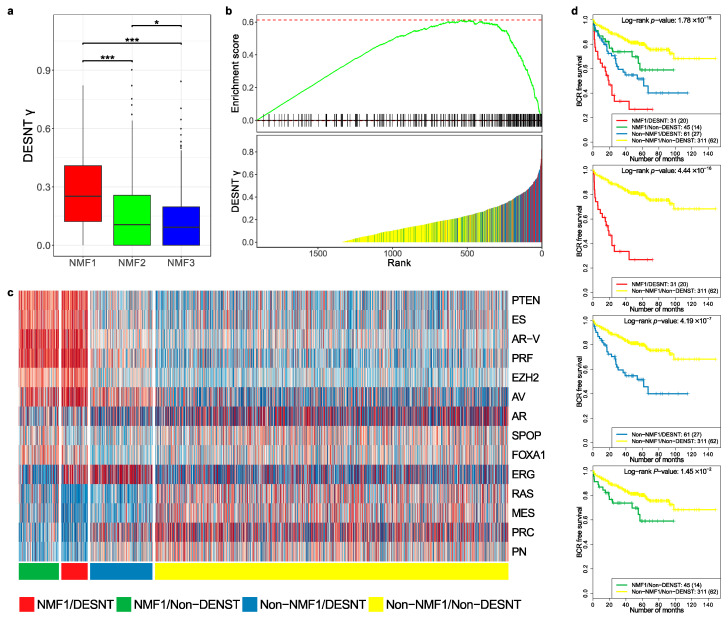
Comparison of DESNT and non-negative matrix classifications. (**a**) Distribution of DESNT γ for cancers assigned to NMF1, NMF2 and NMF3. (**b**) Gene Set Enrichment Analysis. Cancers were ranked according to DESNT γ (Lower Panel). The enrichment for cancers assigned to the NMF1 high-risk group (vertical lines) is shown (Upper Panel). (**c**) Pathway activation profiles for each cancer arranged according to DESNT and NMF1 subgroup status. The key is shown at the bottom of the figure. (**d**) Kaplan–Meir plots for the different cancer categories. The outcome used is time to biochemical recurrence post prostatectomy. * ≤0.05; ** ≤0.01; *** ≤0.001.

We wished to further investigate the relationship between the DESNT and NMF1 poor prognosis groups. To achieve this, the MSKCC, CancerMap, CamCap, and TCGA datasets were combined and DESNT was plotted as a continuous variable (DESNT γ), as described in Luca et al. [27]. DESNT γ was significantly higher in NMF1 cancers compared to NMF2 and NMF3 cancers (Figure 4a) and the results of Gene Set Enrichment Analysis (GSEA) analysis show a highly significant association (*p* < 1 × 10^−6^), giving an enrichment score of 0.61 (Figure 4b).

We next calculated pathway status (*z*-scores, as shown in Figure 2b) for all samples in the MSKCC, CancerMap, CancerMap and TCGA datasets and grouped the samples according to NMF1 and DESNT status. The results are shown in Figure 4c. Cancers assigned both as DESNT and NMF1 had the strongest association with time to progression (Figure 4d, *p* = 4.4 × 10^−16^, Log-rank test) followed by DESNT-non-NMF1 cancers (*p* = 4.19 × 10^−7^) and non-DESNT-NMF1 cancers (*p* = 1.45 × 10^−2^). Membership of DESNT accounted for 36% (31/86) of NMF1 cancers in this series but 59% (31/45) of its PSA failures. Notably, activation of the PTEN, ES, AR-V, PRF and EZH2 pathways, a feature of NMF1 cancers, was not present in DESNT-non-NMF1 cancers.

We conclude from these studies that NMF1 and DESNT are overlapping but distinct cancer categories.

## 4. Discussion

A number of critical observations arise from the presented studies. Signatures derived by comparisons of expression profiles to clinical features (e.g., to Gleason Score and to PSA failure) exhibited only modest overlaps in gene lists. This was exactly as predicted from previous analyses of breast cancer datasets [29]. When normal cells change into a cancer cells or when the clinical state of a cancer is altered, many thousands of genes may exhibit altered expression levels and multiple control pathways modulated. Based only on the analyses performed to identify these biomarkers, it is not possible to determine whether the genes identified are central to cancer development or represent secondary events. Nonetheless, when biomarker analyses are combined with additional studies, useful individual genes are highlighted. For example, HOXB13 was identified in expression array studies as a gene highly upregulated in prostate cancer [48] but its central importance to cancer development was not established until the analyses of cancer families were performed [49]. AMACR was first identified as a gene upregulated in three of four expression array datasets from prostate cancer, but its importance as a cancer marker was not recognised until immunohistochemical studies of tissue sections were performed [50].

When significant overlaps do occur between predictive gene lists developed using entirely different approaches, it is our belief that this indicates genes fundamental for controlling cancer progression. The observation that HES6-signature reported by Ramos-Montoya et al. [22] overlaps with the PCS1 [26] and Prolaris [19] signatures supports this view. HES6 drives castration-resistant tumour growth by enhancing the transcriptional activity of the androgen receptor, while the Prolaris signature contains many genes known to be critical for cell cycle control—both processes already known to be essential for prostate cancer growth. A second overlap occurred between downregulated DESNT genes [23,27] and a set of genes overexpressed in PCS3 [26]. We propose that genes from these two categories are also involved in processes fundamental to the development of prostate cancer. The precise mechanism is currently unknown, although possible but different models were suggested both by Luca et al. [23] and by You et al. [26].

Support for this model was obtained from analyses of the impact of DESNT and SIG-HES6 signatures on clinical outcome. When patients were designated as poor prognosis by DESNT and by one or more of the SIG-HES6 signatures (Prolaris, PCS1, Ramos-Montoya et al.), a considerably worse outcome was observed compared with use of DESNT or SIG-HES6 signatures alone, consistent with interaction. This observation also has implications for patient management, indicating that use of DESNT classification together with, for example, the Prolaris biomarker or the Ramos-Montoya et al. biomarker could greatly increase the ability to predict whether a patient with organ-confined prostate cancer will progress following treatment. This would allow targeting of treatment to the patients who need it hence avoiding the side effects, including impotence, of unnecessary treatment in men with indolent disease.

The overlapping signature DESNT, PCS3, HES6, Prolaris and PCS3 are all derived using unsupervised approaches or by investigation of biological function. It is of interest that not all signature derived using these approaches demonstrated highly significant overlaps. The derivation of the 70-gene biomarker proposed by Walker et al. [25] represent an interesting case. A 222-gene signature was originally generated using an unsupervised approach. The 70-gene signature represents a subset of these genes derived by a combination of unsupervised and supervised approaches. We failed to observe highly significant overlaps involving this signature (Figure S3). Additional signatures involving unsupervised steps that failed show gene overlaps includes those derived by Lalonde et al. [28] and by Ross Adams et al. [24].

We provide evidence that classifications based on NNMF analysis of control pathways and the DESNT classification are overlapping but distinct. The methods of clinical applications of the two tests are also different. Assignment to the PCS1 poor prognosis category is based on the use of a classifier of 37-gene classifier [26] selected from genes’ differential expression between the PCS1, PCS2, and PCS3 groups. In contrast, the poor prognosis DESNT signature is only considered to be present in part of the cancer, with the exact proportion (or DESNT γ) calculated by LPD carried out on genes with the most variable levels of expression across samples [23,27]—the DESNT gene signature itself cannot be used to calculate outcome. Once calculated, the proportion of DESNT cancer can be used in a nomogram together with clinical variables to estimate likelihood of PSA failure [27]. Additionally, PCS1 and PCS3 had been assigned as having, respectively, luminal and basal phenotypes based on the expression of a set of 12 genes [26]. In contrast, we failed to find differential expression of these same genes when comparing DESNT and non-DESNT cancers (result not shown).

An important finding is that all of the highly overlapping signatures predicting poor outcome appear to be sampling from the same high-risk cancer group: the SIG-HES6-and SIG-DESNT groups of signatures are not detecting entirely separate groups of high-risk cancers. This result as well as the observed interactions between DESNT and SIG-HES6 signatures in identifying patients with poor outcome are both consistent with a model where underlying molecular processes represented by SIG-HES6 and SIG-DESNT interact, leading to aggressive disease. This observation may have relevance to approaches for therapeutic targeting. In the clinical setting, the HES6-associated signature can be pharmacologically targeted by inhibition of PLK1 with restoration of sensitivity to castration [22]. For the DESNT signature, many of the genes with downregulated expression in prostate cancer are hypermethylation [23], indicating that 5-azacytidine that could be used to enhance gene expression. Thus, a prediction of the current studies is that the combined use of inhibitors of HES6 function, androgen withdrawal and strategies for gene re-expression, would synergise in preventing the growth of castration-sensitive prostate cancer. Our results also have an implication for the use of biomarkers in general since the use of DESNT together with a SIG-HES6 biomarker may represent a much more effective method for detecting patients with aggressive disease.

## 5. Conclusions

To our knowledge this is the first publication to systematically analyze the relationships between multiple distinct prognostic signatures for prostate cancer. We start with the hypothesis that highly significant overlaps between signatures derived using different approaches indicates genes and processes fundamental to prostate cancer progression; leading to the identification of two sets of overlaps designated SIG-HES6 and SIG-DESNT. First, we conclude that our results support a model whereby SIG-HES6 and SIG-DESNT genes co-operated to cause cancer progression. Secondly, consistent with this model, the use of a SIG-HES6 signature in combination with DESNT provides a much better predictor of poor outcome than the use of either alone. Thirdly, for the drug treatment of patients we predict a synergy between (i) inhibitors of HES6 function, and (ii) agents, such as 5-azacytidine, that can induce re-expression of DESNT genes.

## Figures and Tables

**Table 1:** Prognostic and Classification gene signatures. Abbreviations are as follows: A, signature discovered by association with clinically distinct states, B, signature representing a biological function; U, signature identified by unsupervised approach; LPD, Latent Process Decomposition; OAS-LPD, One Added Sample-LPD; HCA, Hierarchical Cluster Analysis; ADT, Androgen Deprivation Therapy; NNMF, Non-Negative Matrix Factorisation; RP, Radical Prostatectomy; PSA Prostate Specific Antigen.

Citation	Year	Genes	Type	Discovery Method	Name
Agell et al.	2012	12	A	Association to Gleason	-
Bibkova et al.	2007	16	A	Association to Gleason	-
Bismar et al.	2006	12	A	Benign vs. Cancer vs. Metastases	-
Cuzick et al.	2011	31	B	Cell Cycle Genes	Prolaris
Erho et al.	2013	22	A	Cancers with Different Progressions	DECIPHER
Glinksy et al.	2004	11	A	PSA Failure vs. No-failure	-
Irshad et al.	2013	19	B	Aging Genes Altered in Indolent Cancer	-
Klein et al.	2014	17	A	Association with Outcome	OncotypeDX
Lalonde et al.	2014	276	U	Genes within Copy Number Changes	-
Long et al.	2011	13	A	PSA failure vs. No failure	-
Luca et al.	2017	45	U	LPD	DESNT
Luca et al.	2020	49	U	OAS-LPD	OAS-DESNT
Mo et al.	2018	93	B + A	Stroma association to metastasis	-
Planche et al.	2011	48	A	Normal vs. Tumour differential gene expression in stroma	-
Rajan et al.	2014	7	A	Before and After ADT	-
Ramos-Montoya et al.	2014	222	B	Genes Controlled by HES6	-
Ramaswamy et al.	2003	17	A	Metastases vs. Primary	-
Ross-Adams et al.	2014	100	U	Clustering of Variable Genes	-
Sharma et al.	2013	16	B	Androgen Receptor Regulated	-
Singh et al.	2002	29	A	Associated with Gleason	-
Varambally et al.	2005	44	A	Metastases vs. Primary	-
Walker et al.	2017	70	U + A	HCA and PLS Regression *	-
Wu et al.	2013	32	A	Associated with Outcome	-
You et al.	2016	428	U	NNMF of Control Pathways	PCS1, PCS2, PCS3
Yu et al.	2007	7	B	Polycomb Repression Signature	-

* Applied HCA for subgroup identification and partial-least-squares regression for signature development. All studies cited are listed in the reference section.

## Supplementary Materials

The following are available online at https://www.mdpi.com/2073-4425/11/7/802/s1. Figure S1: MDS (Multi-Dimensional-Scaling) decomposition of the expression profiles of the DISC cohort, Figure S2: The out-of-bag(OOB) performance of NNMF random forest classifier on the DISC cohort, Figure S3: Overlaps between the gene signatures considered in this study, Figure S4: Genes identified as subtype-enriched by You et al., Figure S5: Comparison of altered genes identified by non-negative matrix factorization (NNMF) of control pathways; Table S1: Repetition of the non-negative matrix factorization (NNMF) categorisation of prostate cancer described by You et al.; Supplementary materials: Data S1: List of biomarker and signature gene used in Figure S3, Data S2: Overlaps between the DESNT, OAS-DESNT, and PCS3 gene signatures, Data S3: Overlap gene signatures for the Prolaris, PCS1, and Ramos-Montoya et al. (HES6) gene signatures; Data S4: Comparisons of differentially expressed genes, Data S5: Overlaps between the DESNT, OAS-DESNT, and NMF3 gene signatures.
